# Phosphorylated eIF2α predicts disease-free survival in triple-negative breast cancer patients

**DOI:** 10.1038/srep44674

**Published:** 2017-03-15

**Authors:** Liang Guo, Yayun Chi, Jingyan Xue, Linxiaoxi Ma, Zhiming Shao, Jiong Wu

**Affiliations:** 1 Department of Breast Surgery, Key Laboratory of Breast Cancer in Shanghai, Fudan University Shanghai Cancer Center, Shanghai 200032, PR China; 2 Department of Oncology, Shanghai Medical College, Fudan University, Shanghai 200032, PR China; 3 Collaborative Innovation Center for Cancer Medicine, Guangzhou 510060, China

## Abstract

Phosphorylated eukaryotic translation initiation factor 2α (p-eIF2α), which functions as a marker of endoplasmic reticulum stress, has been reported to be associated with patient prognosis in various cancers. However, little is known about the prognostic value of p-eIF2α in breast cancer, particularly in different breast cancer subtypes. An immunohistochemistry screen for p-eIF2α was performed using a tissue microarray containing 233 tumors and paired peritumoral tissues from female patients diagnosed with breast cancer. The staining results were scored semiquantitatively, and the p-eIF2α expression level in breast cancer and its potential prognostic value were investigated. In this retrospective cohort study, we found that p-eIF2α levels were significantly upregulated in breast cancer (P < 0.001). p-eIF2α level was negatively correlated with lymph node status (P = 0.039). Survival analysis by Kaplan–Meier estimation and Cox regression showed that p-eIF2α level was correlated with better disease free survival (P = 0.026) and served as an independent prognostic factor (P = 0.046) in patients with triple-negative breast cancer. Our study revealed that p-eIF2α was upregulated in breast cancer and represented a novel predictor of prognosis in patients with triple-negative subtype.

The endoplasmic reticulum (ER) is a eukaryotic cell organelle responsible for lipid biosynthesis, intracellular Ca^2+^ homeostasis, and protein folding and transport. The ER protein-folding environment can be disrupted by numerous events, including nutrient fluctuations, as well as environmental, physiological, and pathological damage. The protein misfolding and accumulation that results from such disruption is termed ER stress^1^. The unfolded protein response (UPR) is a collection of signaling pathways that respond to unfolded proteins in the ER lumen^2^. Extensive evidence suggests that ER stress and UPR activation are involved in the development of several cancer types and play important roles in every aspect of cancer, including tumor initiation, development and progression.

The UPR comprises three principal parallel branches: the PKR-like ER kinase (PERK)–eukaryotic translation initiation factor (eIF) 2α pathway; the inositol-requiring protein 1α (IRE1α)–X-box binding protein 1(XBP1) pathway; and the activating transcription factor (ATF) 6α pathway^3^. Although previous research indicated that XBP1 mRNA splicing increased in human triple-negative breast cancers (TNBCs)^4^, the main role of IRE1α–XBP1 signaling has been more extensively investigated in multiple myeloma because the pathway is involved in regulating mature B cell differentiation^5^,^6^. Some studies have suggested that ATF6α can regulate its target genes, which are involved in the development of hepatocarcinoma^7^. The function of the PERK–eIF2α pathway in tumors is still uncertain; signaling via this pathway may induce either survival or apoptosis of tumor cells upon ER stress, and can either promote or inhibit malignant transformation.

eIF2 comprises three subunits: α, β, and γ. The α-subunit of eIF2, eIF2α, can be phosphorylated on Ser51, thereby effectively reducing the level of active eIF2. In this way, p-eIF2α can significantly inhibit mRNA translation initiation^8^ and global protein synthesis^9^. Recently, evidence has mounted to demonstrate that p-eIF2α upregulation is associated with tumor development and progression^10^,^11^,^12^. Conversely, other studies have demonstrated that p-eIF2α has a potential protective effect^13^,^14^,^15^. Thus, the detailed functions of p-eIF2α in tumors remain unclear. In recent years, the role of ER stress and UPR activation in the development of breast cancer, which has variable prognosis based on distinctive molecular subtyping, has attracted increasing attention. However, the prognostic value of p-eIF2α in breast cancer is not yet known.

In this study, we investigate whether p-eIF2α could serve as a prognostic biomarker in breast cancer, with a particular focus on differences between different molecular subtypes; and provide evidence for a new therapeutic target in breast cancer.

## Results

### Patient characteristics

Initially, 243 female breast cancer cases were included in this study. Of these patients, 10 cases experienced tissue loss after IHC staining. The remaining 233 cases were included in the subsequent analysis. The excluded cases were not substantially different in all major prognostic factors compared with those with available information. The clinicopathological characteristics of the patients with available information in this study are summarized in Table 1. There are 84(36.05%) luminal breast cancer patients, 86(36.91%) Her2+ breast cancer patients and 63(27.04%) triple-negative breast cancer patients respectively. After a mean follow-up time of 60.77 months, 38 of the 233 patients experienced disease recurrence or tumor metastasis.

### p-eIF2α is upregulated in breast cancer

**Figure 1:**
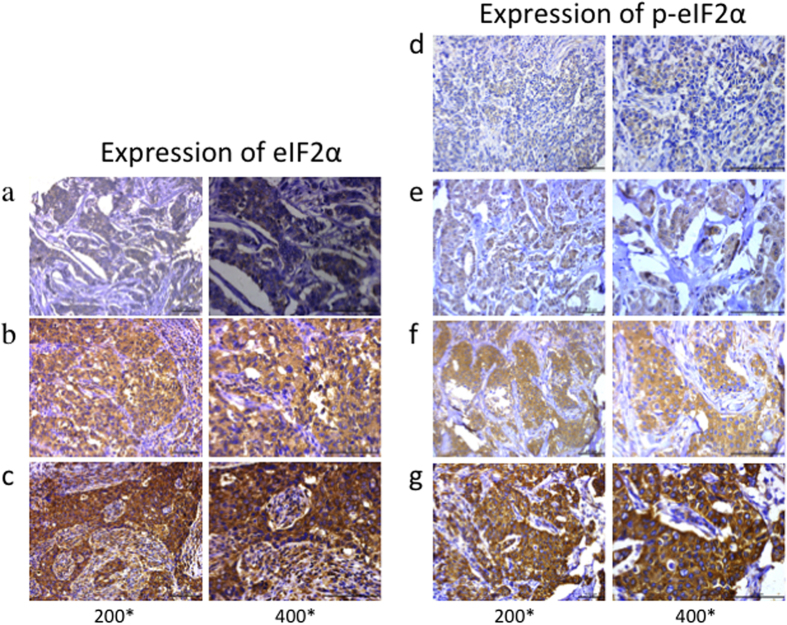
Total eIF2α and p-eIF2α expression profiles and scoring in breast cancer tissues. Total eIF2α was evaluated in breast cancer and divided into three groups. Representative images of tissue scored as (**a**) 1, (**b**) 2, and (**c**) 3. p-eIF2α immunostaining was divided into four groups. Representative images of tissue scored as (**d**) 0, (**e**) 1, (**f**) 2 and (**g**) 3. Left photomicrographs, 200× magnification; right photomicrographs, 400× magnification.

**Figure 2:**
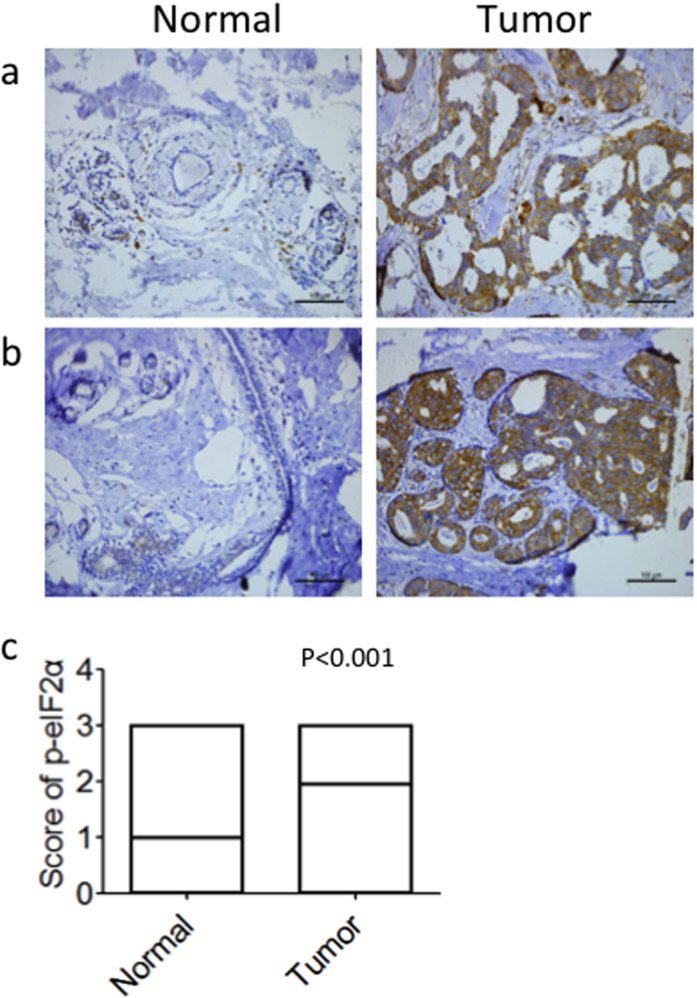
Comparison of p-eIF2α levels between tumor and peritumoral tissues in patients with breast cancer. Representative staining of p-eIF2α in peritumoral and tumor tissues in two patients is shown. (**a**,**b**) Photomicrographs, 200× magnification. (**c**) Wilcoxon signedrank comparison of p-eIF2α levels between 233 pairs of tumor and peritumoral tissues.

Representative staining of total eIF2α and p-eIF2α is shown in Fig. 1. We detected p-eIF2α expression in tissue microarrays that included specimens from 233 patients with breast cancer (paired tumor and peritumor tissues). p-eIF2α was expressed mainly in the cytoplasm (Fig. 2a and b) in both the tumor and peritumor tissues. High expression of p-eIF2α was detected in 60.1% of tumor tissue specimens (Table 1), compared with in 31.2% of peritumor tissue specimens (data not shown). Paired comparison demonstrated that p-eIF2α was significantly upregulated in breast cancer (P < 0.001, Fig. 2c).

### p-eIF2α expression pattern in patients with breast cancer

We next assessed the relationship between p-eIF2α and clinicopathological characteristics. We found that there was no correlation between p-eIF2α expression and age, menopausal status, histological grade, tumor size or lymphatic vascular invasion in patients with breast cancer. We also found no correlation between p-eIF2α expression and estrogen receptor (ER), progesterone receptor (PR), Her2, Ki67 status, or molecular subtype. However, p-eIF2α expression was negatively correlated with lymph node status (P = 0.039, Table 1).

We also investigated the relationship between p-eIF2α expression and clinicopathological characteristics in patients with TNBC and found no correlations (Table 2).

### p-eIF2α predicts disease-free survival in patients with TNBC

**Figure 3:**
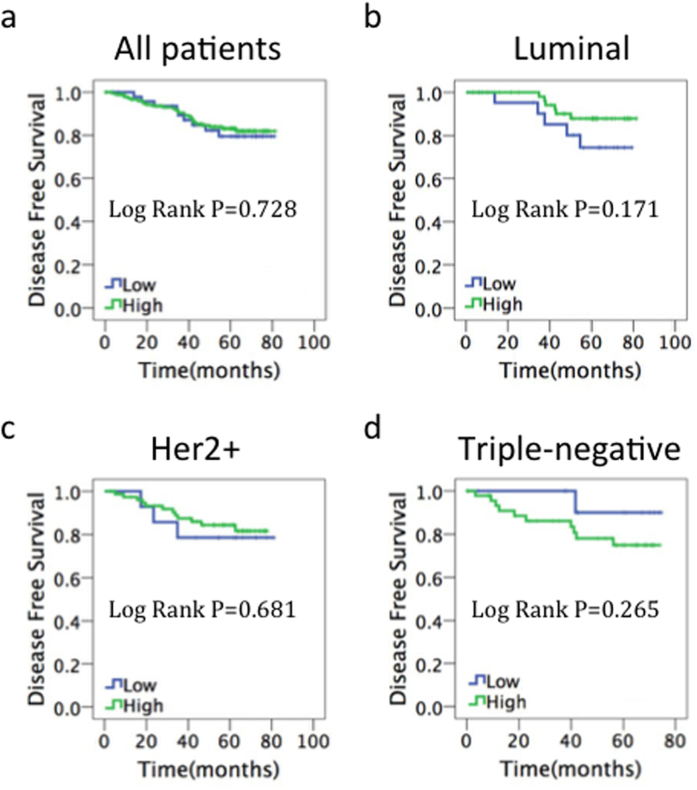
Prognostic value of total eIF2α in breast cancer. Kaplan–Meier survival curves of disease-free survival for (**a**) all patients, (**b**) patients with estrogen receptor-positive/human epidermal growth factor receptor 2 (HER2)-negative disease, (**c**) patients with HER2-positive disease, (**d**) patients with estrogen receptor-negative/HER2-negative disease.

**Figure 4:**
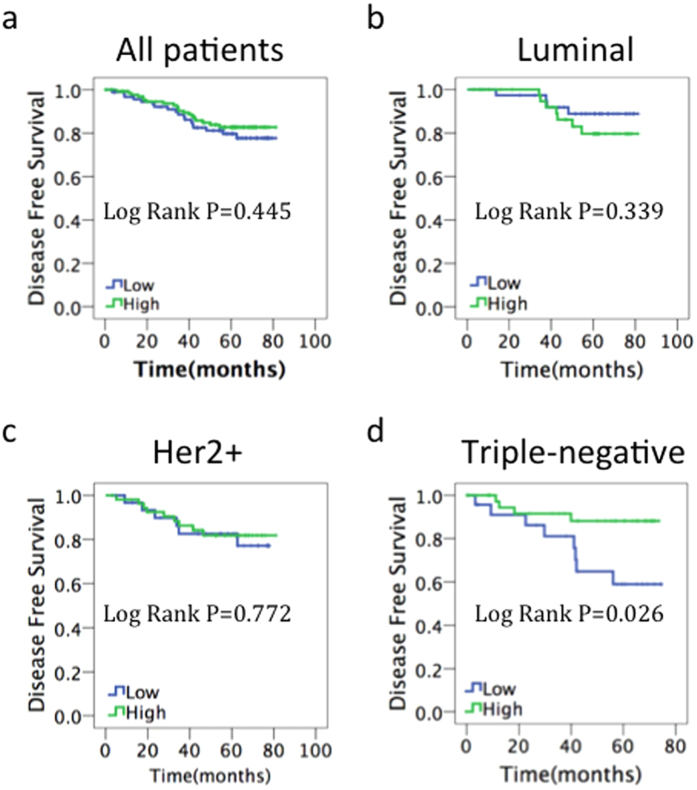
Prognostic value of p-eIF2α in breast cancer. Kaplan–Meier survival curves of disease-free survival for (**a**) all patients, (**b**) patients with estrogen receptor-positive/human epidermal growth factor receptor 2 (HER2)-negative disease, (**c**) patients with HER2-positive disease, (**d**) patients with estrogen receptor-negative/HER2-negative disease.

For the prognostic evaluation, we examined total eIF2α and p-eIF2α levels and their association with disease-free survival (DFS) and overall survival (OS). We did not find any correlation between total eIF2α levels and DFS (Fig. 3). There was also no significant prognostic effect for p-eIF2α in the global population (Fig. 4a), in patients with the luminal breast cancer subtype (ER-positive and Her2-negative) (Fig. 4b), or in the Her2-positive subgroup (Fig. 4c). However, p-eIF2α level was found to be strongly related to DFS by Kaplan–Meier survival analysis in the triple-negative subgroup (P = 0.026, Fig. 4d). Median DFS was 67.5 months in patients with high p-eIF2α expression and 57.3 months in those with low p-eIF2α expression. As a result of short follow-up time and less death-related events, we did not find any correlation between total eIF2α and p-eIF2α levels with OS (Supplementary Figs 1 and 2).

In univariate analysis, correlations between DFS and each clinicopathological parameter were examined for TNBC. Only p-eIF2α demonstrated an association with DFS (HR = 0.280, 95% CI: 0.084–0.930, P = 0.038). In multivariate analysis, p-eIF2α remained statistically significant (HR = 0.199, 95% CI: 0.041–0.973, P = 0.046)(Table 3). These data indicate that p-eIF2α could serve as an independent prognosis marker in TNBC.

## Discussion

Previous studies have reported detection of high p-eIF2α expression levels in tumor samples compared with in matched noncancerous tissues, in cancers including bronchioloalveolar carcinomas of the lung^16^, Hodgkin lymphoma^17^, gastrointestinal carcinomas^18^ and malignant melanoma^19^. Consistent with these previous findings, in this study, we found that p-eIF2α levels are also significantly higher in breast cancer than in peritumor tissues. Generally speaking, p-eIF2α levels reflect the severity of ER stress in tumors, indicating that ER stress plays an important role in the initiation of tumor formation. However, in some tumors, such as human osteosarcoma, the opposite relationship is observed: levels of p-eIF2α are lower in cancerous tissue than in normal tissue^20^, which reflects the heterogenicity of different tumors.

Many attempts have been made to identify an association between ER stress markers and prognosis in breast cancer. XBP1 promotes TNBC progression via the hypoxia-inducible factor 1α (HIF1α) pathway^4^. Additionally, XBP-1 expression and splicing are associated with clinical outcome in endocrine-treated breast cancer, depending on the XBP-1 isoform^21^. Cell surface glucose-regulated protein 78, which is an ER molecular chaperone and a major UPR target, predicts worse disease-free survival; while overexpression of C/EBP homologous protein (CHOP), a pro-apoptotic transcription factor that is activated during UPR, correlates with better survival in patients with breast cancer^22^. Our findings, which demonstrated prognostic value of p-eIF2α in breast cancer, are partially consistent with this previous research, because CHOP is also involved in the PERK-p-eIF2α signaling pathway and predicts better DFS in patients with breast cancer. However, p-eIF2α was only identified as a prognostic indicator in patients with the TNBC subtype in this study. We speculate that this is because TNBC has underlying heterogeneity compared with the luminal and Her2-positive subtypes. The possible mechanisms underlying the prognostic value of p-eIF2α still need to be explored.

The prognostic value of p-eIF2α in tumor types other than breast cancer is still unclear, as described above. p-eIF2α inhibits the synthesis of large amounts of proteins that is a necessary part of the tumorigenesis process. However, it may lead to an increase in ATF4, CHOP and other factors^23^,^24^, all of which may aid or impede tumor progression depending on the extent of the stress. We suggest that p-eIF2α has a predominantly inhibitory effect on tumor growth in TNBC. As we expected, higher p-eIF2α expression was associated with lower tumor invasion of lymph nodes. Our study had several limitations, including fewer patients with TNBC and shorter follow-up time for the patient cohort.

Of the various breast cancer subtypes, TNBC has the greatest need for improved therapies because it is clinically aggressive and usually relapses and progresses in a short time. Although sensitive to conventional chemotherapy^25^, TNBC remains the breast cancer subtype with the worst patient prognosis. Moreover, TNBC therapy remains challenging because of the underlying heterogeneity of TNBC and the lack of predictive biomarkers and effective therapeutic targets. In this study, we illustrated that p-eIF2α may be a potential target for the treatment of TNBC. Aktas *et al*. previously identified three small molecular weight compounds that induce eIF2α phosphorylation, for use in cancer therapy^26^. However, therapeutic targeting of p-eIF2α remains challenging because of the dual function of p-eIF2α, which could result in severe side effects.

In this study, we demonstrated that p-eIF2α predicted disease-free survival and could serve as an independent prognostic biomarker in TNBC. This finding suggests that evaluating p-eIF2α expression in breast cancer may have a potential clinical application, by providing additional information for oncologists when individualizing cancer management.

## Methods

### Patients and specimens

Specimens and data were collected from 233 female patients who were diagnosed with stage I to III breast carcinoma at the department of Breast Surgery in Fudan University Shanghai Cancer Center (FDUSCC, Shanghai, P. R. China). Each case was given a specific identifier and linked to a database containing clinicopathological data. The pathological data, including ER, PR, Her2 and Ki67 status, were assessed by FUSCC pathologists using the ASCO breast cancer guidelines. The study was approved by the Ethics Committee of FUSCC, and written informed consent was signed by each patient. All methods were performed in accordance with the relevant guidelines and regulations.

### Immunohistochemistry and tissue microarray scoring

Tissue microarrays containing 233 breast cancer tissues and paired peritumoral tissues were constructed from formalin-fixed and paraffin-embedded samples. The tissue microarrays were first deparaffinized in xylene and rehydrated in a graded alcohol series, then boiled with 10 mmol/L citrate buffer (pH 6) for 15 min and pre-incubated in blocking solution (10% normal goat serum) for 1 h at room temperature. The EnVision two-step method and a DAB Color Kit (Gene Tech Company Limited, Shanghai, China) were used to stain the target molecule. A mouse anti-human monoclonal antibody against eIF2α (1:250, Abcam) and a rabbit anti-human monoclonal antibody against p-eIF2α (1:500, Abcam) were used.

The expression of total eIF2α and p-eIF2α in the immunohistochemically stained specimens was evaluated by two professional pathologists concurrently and assigned scores according to the intensity of the staining (0, negative; 1, weak; 2, moderate; 3, strong) and the percentage of cells stained (1, 0 to 10%; 2, 10 to 50%; 3, 50 to 100%), based on the semiquantitative method of Allred *et al*.^27^. As a reason of wide expression of total eIF2α, the classification for the total eIF2α was three groups (weak, moderate and strong). The final total eIF2α and p-eIF2α index was determined based on these two variables; the index was considered high when the scores for both variables were at least two, and low if they were not.

### Statistical analysis

The end point of the study was disease-free survival (DFS). DFS was defined as the time from the date of surgery to the date of first relapse, second primary malignancy or death resulting from any cause. Overall survival (OS) was defined as the time elapsed from the date of surgery to the date of death from any cause or the date of last follow-up. Statistical Package for the Social Sciences software (version 17.0, SPSS Inc, Chicago, IL, USA) was used to analyze all the statistical data. Survival curves were generated using the Kaplan–Meier method and compared using the log-rank test. Hazard ratios (HR) and 95% confidence intervals (95% CI) for the variables were calculated using the Cox proportional hazards model. Univariate and multivariate Cox regression analyses were used to evaluate the significance of various parameters for survival. The associations between p-eIF2α expression and clinicopathological variables were calculated using either χ^2^ tests with continuity correction or Fisher’s exact tests. The Wilcoxon signedrank test was used when comparing paired ranked data.All statistical tests used were two sided, and P < 0.05 was considered significant. All analyses were based on the observed data with the assumption that missing data were randomly distributed.

## Additional Information

**How to cite this article:** Guo, L. *et al*. Phosphorylated eIF2α predicts disease-free survival in triple-negative breast cancer patients. *Sci. Rep.* **7**, 44674; doi: 10.1038/srep44674 (2017).

**Publisher's note:** Springer Nature remains neutral with regard to jurisdictional claims in published maps and institutional affiliations.

## Supplementary Material

Supplementary Figures

## Figures and Tables

**Table 1:** Frequency of clinicopathological characteristics in all breast cancer patients according to p-eIF2α expression.

Characteristics	n	p-eIF2α		P
		Low	High	
**Age (years)**				0.451
<50	87	32(36.8%)	55(63.2%)	
≥50	146	61(41.8%)	85(58.2%)	
**Menopausal status**				0.467
Pre	126	53(42.1%)	73(57.9%)	
Post	107	40(37.4%)	67(62.6%)	
**Pathological subtype**				0.073
IDC	204	77(37.7%)	127(62.3%)	
Others	29	16(55.2%)	13(44.8%)	
**Histological grade**				0.425
Low	124	49(39.5%)	75(60.5%)	
High	86	32(37.2%)	54(62.8%)	
Unknown	23	12(52.2%)	11(47.8%)	
**Stage**				0.392
I and II	182	70(38.5%)	112(61.5%)	
III	51	23(45.1%)	28(54.9%)	
**Tumor size**				0.686
≤2 cm	61	23(37.7%)	38(62.3%)	
2–5 cm	121	47(38.8%)	74(61.2%)	
>5 cm	51	23(45.1%)	28(54.9%)	
**Node status**				0.039
Negative	127	43(33.9%)	84(66.1%)	
Positive	106	50(47.2%)	56(52.8%)	
**LVI**				0.127
Negative	149	54(36.2%)	95(63.8%)	
Positive	84	39(46.4%)	45(53.6%)	
**ER status**				0.852
Negative	116	47(40.5%)	69(59.5%)	
Positive	117	46(39.3%)	71(60.7%)	
**PR status**				0.608
Negative	128	53(41.4%)	75(58.6%)	
Positive	105	40(38.1%)	65(61.9%)	
**HER-2 status**				0.519
Negative	147	61(41.5%)	86(58.5%)	
Positive	86	32(37.2%)	54(62.8%)	
**Ki67 status**				0.707
<20%	94	38(40.4%)	56(59.6%)	
≥20%	80	34(42.5%)	46(57.5%)	
Unknown	59	21(35.6%)	38(64.4%)	
**Molecular subtype**				0.458
Luminal	84	38(45.2%)	46(54.8%)	
Her2+	86	32(37.2%)	54(62.8%)	
Triple-negative	63	23(36.5%)	40(63.5%)	
**Chemotherapy**				0.267
Doxorubicin based	124	55(44.4%)	69(55.6%)	
Taxanes added	82	26(31.7%)	56(68.3%)	
CMF or Xeloda	6	2(33.3%)	4(66.7%)	
None	21	10(47.6%)	11(52.4%)	
**Radiotherapy**				0.703
No	157	64(40.8%)	93(59.2%)	
Yes	76	29(38.2%)	47(61.8%)	

Abbreviations: IDC = invasive ductal carcinoma; Low histological grade = 1 or 2, high histological grade = 3; LVI = lymphatic vascular invasion; ER = estrogen receptor; PR = progesterone receptor; Her2 = human epidermal growth factor receptor-2; Luminal represents Luminal A or Luminal B breast cancer patients; Her2+ represents Her2/Luminal B or Her2+ breast cancer patients. CMF: Cyclophosphamide, Methotrexate and 5-Fluorouracil.

**Table 2:** Frequency of clinicopathological characteristics in triple-negative breast cancer patients according to p-eIF2α expression.

Characteristics	n	p-eIF2α		P
		Low	High	
**Age (years)**				0.743
<50	23	9(39.1%)	14(60.9%)	
≥50	40	14(35.0%)	26(65.0%)	
**Menopausal status**				0.907
Pre	35	13(37.1%)	22(62.9%)	
Post	28	10(35.7%)	18(64.3%)	
**Histological grade**				0.255
Low	25	7(28.0%)	18(72.0%)	
High	38	16(42.1%)	22(57.9%)	
**Tumor size**				0.396
≤5 cm	55	19(34.5%)	36(65.5%)	
>5 cm	8	4(50.0%)	4(50.0%)	
**Node status**				0.157
Negative	40	12(30.0%)	28(70.0%)	
Positive	23	11(47.8%)	12(52.2%)	
**LVI**				0.194
Negative	47	15(31.9%)	32(68.1%)	
Positive	16	8(50.0%)	8(50.0%)	
**Ki67 status**				0.128
<20%	15	3(20.0%)	12(80.0%)	
≥20%	48	20(41.7%)	28(58.3%)	
**Chemotherapy**				0.26
Doxorubicin based	30	8(26.7%)	22(73.3%)	
Taxanes added	27	12(44.4%)	15(55.6%)	
CMF or Xeloda	1	1(100.0%)	0(0.0%)	
None	5	2(40.0%)	3(60.0%)	
**Radiotherapy**				0.128
No	48	20(41.7%)	28(58.3%)	
Yes	15	3(20.0%)	12(80.0%)	

**Table 3:** Univariate and multivatiate analysis of factors for DFS in triple-negative breast cancer patients.

Variable	Category	Univariate analysis			Multivariate analysis		
		HR	95% CI	P-value	HR	95% CI	P-value
Age (years)	≥50/<50	1.03	0.310–3.422	0.962	0	0–2.813E + 98	0.945
Menopause	Positive/Negative	0.674	0.203–2.240	0.519	0	0–1.056E + 98	0.939
LVI	Positive/Negative	1.389	0.418–4.618	0.592	0.555	0.116–2.657	0.462
Histological grade	High/Low	1.392	0.419–4.628	0.589	1.371	0.319–5.891	0.671
Tumor size	>5 cm/≤5 cm	1.119	0.245–5.123	0.885	0.263	0.029–2.432	0.24
Node status	Positive/Negative	2.465	0.782–7.773	0.124	2.241	0.241–20.791	0.478
Ki67 status	>20%/≤20%	0.56	0.168–1.869	0.346	0.259	0.050–1.341	0.107
Chemotherapy	Others/Doxorubicin based	1.9	0.572–6.310	0.295	1.011	0.201–5.085	0.989
Radiotherapy	Yes/No	1.666	0.501–5.539	0.405	3.536	0.560–22.318	0.179
p-eIF2α	High/Low	0.28	0.084–0.930	0.038	0.199	0.041–0.973	0.046
