# Supplementary material for: Phosphorylated eIF2α predicts disease-free survival in triple-negative breast cancer patients
Source: Sci Rep. 2017 Mar 15;7:44674. doi: 10.1038/srep44674 (PMC5353635; doi:10.1038/srep44674)
Supplement: Supplementary Figures [file srep44674-s1.pdf]

Phosphorylated eIF2α predicts disease-free survival in triple-negative breast cancer patients

**Liang Guo[1,2#] Yayun Chi [1,2#] Jingyan Xue[1,2#] Linxiaoxi Ma[1,2] Zhiming Shao[1,2] Jiong Wu[1,2,3]*[**]

1. Department of Breast Surgery, Key Laboratory of Breast Cancer in Shanghai, Fudan University Shanghai Cancer Center，Shanghai 200032,PR China.

2. Department of Oncology, Shanghai Medical College, Fudan University, Shanghai 200032, PR China.

3. Collaborative Innovation Center for Cancer Medicine, Guangzhou 510060, China.

*To whom correspondence may be addressed.
Jiong Wu, MD& PhD, Department of Breast Surgery, Key Laboratory of Breast Cancer in Shanghai, Fudan University Shanghai Cancer Center, Department of Oncology, Shanghai Medical College, Fudan University，Shanghai. 270 Dongan Rd. Shanghai 200032, China. E-mail: wujiong1122@vip.sina.com

# These authors contributed equally to this work.

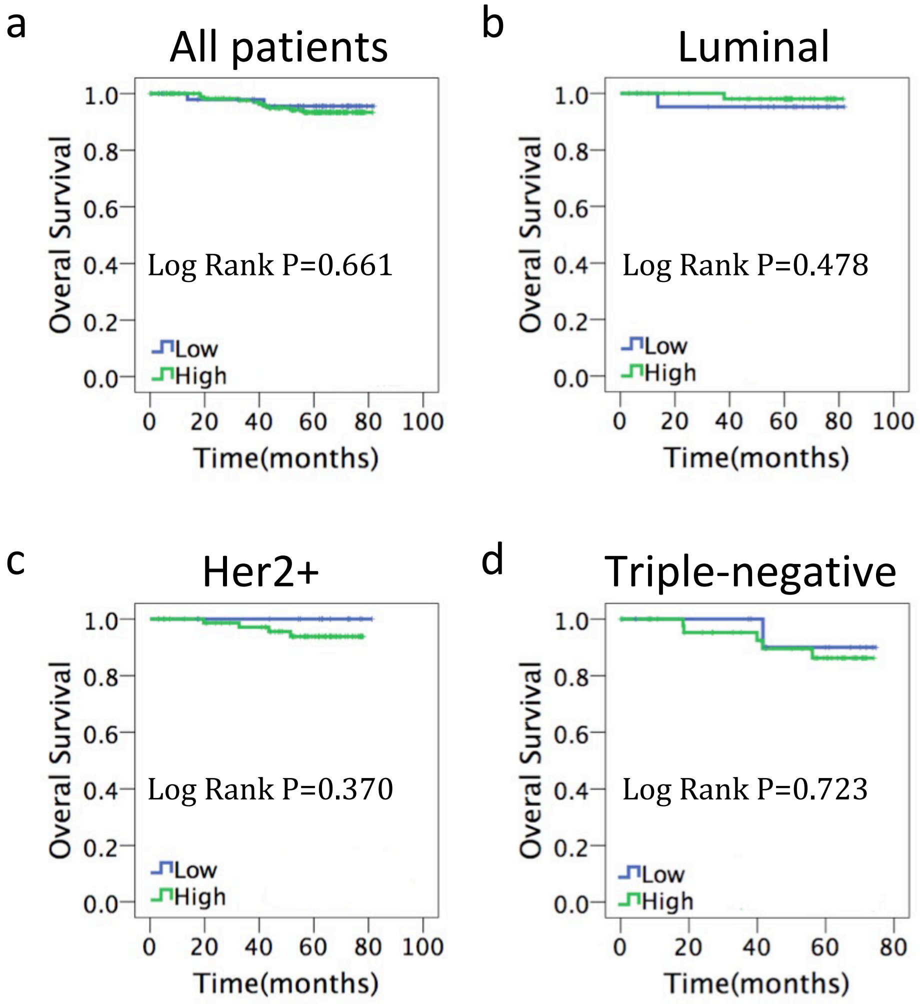

Supplementary Figure.1 Prognostic value of total eIF2α in breast cancer. Kaplan–Meier survival curves of overall survival for (a) all patients, (b) patients with estrogen receptor-positive/human epidermal growth factor receptor 2 (HER2)-negative disease, (c) patients with HER2-positive disease, (d) patients with estrogen receptor-negative/HER2-negative disease.

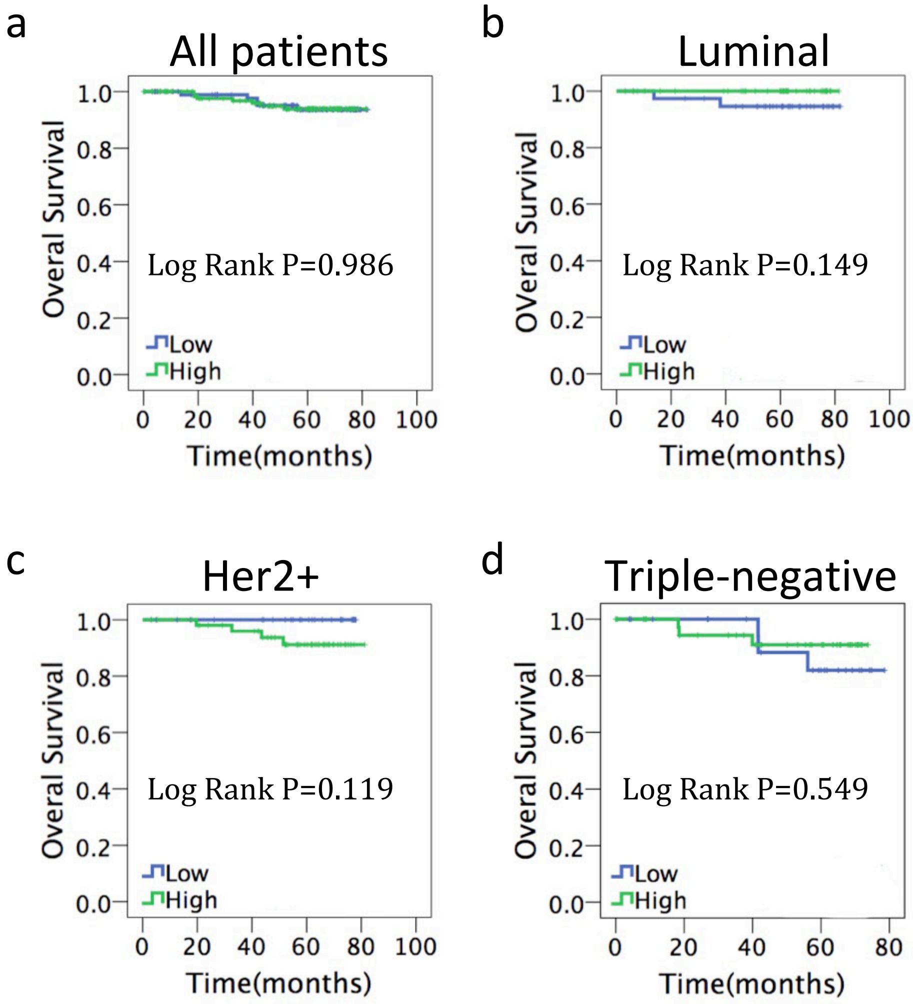

Supplementary Figure.2 Prognostic value of p-eIF2α in breast cancer. Kaplan–Meier survival curves of overall survival for (a) all patients, (b) patients with estrogen receptor-positive/human epidermal growth factor receptor 2 (HER2)-negative disease, (c) patients with HER2-positive disease, (d) patients with estrogen receptor-negative/HER2-negative disease.
